# Factors associated with help-seeking for urinary symptoms among Chinese patients with bladder cancer: A qualitative study

**DOI:** 10.1016/j.apjon.2026.101001

**Published:** 2026-07-01

**Authors:** Bin Wang, Jun Zou, Lingling Gao, Fang Lou, Ziyi Qi, Yun Dai

**Affiliations:** a Department of Nursing, The First Affiliated Hospital, Zhejiang University School of Medicine, Hangzhou, China; b The First Affiliated Hospital of Soochow University, Suzhou, China

**Keywords:** Bladder cancer, Help-seeking behavior, Nursing care, Qualitative study

## Abstract

**Objective:**

To investigate factors associated with help-seeking for urinary symptoms among Chinese patients with bladder cancer.

**Methods:**

A qualitative descriptive research design was employed, involving semi-structured interviews with patients diagnosed with bladder cancer. Content analysis was used to analyze the data.

**Results:**

A total of 19 patients were interviewed . Four key themes were identified: (1) Factors influencing initial symptom appraisal, with three subthemes: attribution of urinary symptoms to non-threatening causes, lack of awareness of hematuria as a cancer sign, andillusion of self-healing caused by painless and intermittent symptoms; (2) Factors sustaining inaction, with three subthemes: masculine endurance, prioritizing social roles, age-related fatalism; (3) Factors converting inaction into action, with three subthemes: symptom persistence or worsening, social support, authoritative advice; (4) Factors that serve as sociocultural and contextual moderators, with four subthemes: financial constraints, festival taboos, the digital divide in healthcare, the warning effect of others’ illness experiences.

**Conclusions:**

Help-seeking behavior among Chinese patients with bladder cancer is influenced by gaps in symptom awareness and appraisal, cultural-psychological barriers, and contextual moderators. Targeted interventions could focus on enhancing risk awareness of hematuria, addressing endurance norms and age-related fatalism, strengthening family and social support, and bridging the digital divide, with the aim of supporting timely help-seeking.

## Introduction

Bladder cancer is one of the most common malignant tumors of the urinary system. According to GLOBOCAN 2022 estimates, approximately 614,000 new cases of bladder cancer and 220,000 deaths occurred worldwide.^1^ Additionally, the postoperative one-year recurrence rate for non-muscle-invasive bladder cancer ranges from 15% to 61%, and the cumulative five-year recurrence rate is as high as 31% to 78%.^2^ Due to the high recurrence rate, patients require long-term follow-up, making bladder cancer one of the most expensive cancers in terms of medical expenditure.^3^ Therefore, bladder cancer is an urgent and significant public health issue.

Painless visible hematuria is the most common initial symptom of bladder cancer, occurring in approximately 80% of patients.^4^ However, hematuria is often misinterpreted as being indicative of more common benign conditions, such as urinary tract infections, urolithiasis, benign prostatic hyperplasia, or age-related physiological changes.^5^ This frequent misattribution to non-malignant causes leads to significant delays in patients' help-seeking behavior. Help-seeking behavior for cancer symptoms is the process of making an informed decision to access healthcare upon detecting potential symptoms. It is mostly defined based on the concept of ‘time’ as ‘the interval between symptom detection and the first visit with a healthcare provider’.^6^ Studies have reported that the median time from symptom onset to first medical help for bladder cancer ranges from 60 to 220 days,^7^^,^^8^ substantially longer than that observed for other common malignancies, such as lung cancer.^9^ Studies have shown that early help-seeking for cancer symptoms facilitates timely diagnosis and improves survival outcomes.^10^ In contrast, delayed help-seeking is linked to more severe complications, higher recurrence rates, and increased mortality.^11^ Therefore, it is important to clarify help-seeking behavior among patients with symptoms suggestive of bladder cancer.

Help-seeking behavior for cancer symptoms is not a straightforward decision but a complex behavior shaped by a multitude of factors. A systematic review of help-seeking behavior in gynecological cancer patients identified three key factor categories: patient-related, emotional, and practical.^12^ Another meta-analysis highlighted psychological factors, including symptom knowledge, interpretation, and cancer beliefs.^13^ A systematic review of psychosocial factors on cancer help-seeking in low- and lower-middle-income countries found that the use of traditional, complementary, and alternative medicine (TCAM) was a key barrier.^14^ Collectively, these studies underscore that cancer symptom help-seeking is influenced by a dynamic interplay of patient-related, psychological, emotional, practical, and sociocultural factors, many of which are context-dependent. This highlights the need to explore how these influences manifest in diverse geographic and cultural settings.

Current research on help-seeking behaviors for cancer symptoms has predominantly focused on breast, gynecological, and lung cancers,^10^^,^^12^^,^^15^ while attention to urological cancer is markedly insufficient, especially in China. Cultural backgrounds and cancer types play a critical role in shaping symptom recognition, expression, and subsequent help-seeking behaviors. Among existing studies related to bladder cancer, most have centered on barriers encountered during diagnosis,^16^ experiences following surgical treatment,^17^ or patients’ lived experiences from diagnosis through recovery.^18^^,^^19^ However, research specifically addressing the period from symptom onset to help-seeking behavior remains relatively scarce.

To address this gap, we employed a descriptive qualitative design to explore the characteristics and associated factors of help-seeking behavior for bladder cancer symptoms within the Chinese sociocultural context. Qualitative description is appropriate when the aim is to provide a straightforward, practice-oriented account of participants’ experiences and the factors shaping those experiences.^20^ This approach facilitated a comprehensive exploration of the experiences of patients with bladder cancer and factors influencing their help-seeking behavior. By clarifying these influences, our study aims to inform the development of culturally appropriate interventions to promote timely help-seeking.

## Methods

### Study design

This study employed a qualitative descriptive research design based on the philosophical tenets of naturalistic inquiry.^21^ Data were collected through semi-structured interviews. In this study, reporting was conducted using the Consolidated Criteria for Reporting Qualitative Research (COREQ).^22^

### Setting and participants

This study was conducted in the Urology Department of The First Affiliated Hospital, Zhejiang University School of Medicine, Hangzhou, China, a large tertiary public hospital in eastern China, from March 2025 to February 2026. The urology department is organized across three campuses and comprises seven wards, providing services to patients from different regions of China.

Purposive sampling was employed to recruit eligible participants based on the inclusion and exclusion criteria, with the aim of achieving broad representation across age groups, educational backgrounds, and disease durations. The inclusion criteria were as follows: (1) diagnosed with bladder cancer according to established diagnostic criteria; (2) age ≥ 18 years; (3) fluency in spoken Mandarin Chinese; (4) able to understand their condition and provide voluntary informed consent. The exclusion criteria included: (1) history or current diagnosis of mental illness or cognitive impairment; (2) current or prior participation in other clinical trials or psychosocial interventions; (3) significant hearing impairment. Among the 21 recruited participants, one dropped out due to severe anemia and weakness caused by long-term hematuria, and another was excluded because of communication difficulties associated with hearing loss.

### Data collection

A semi-structured interview guide (Table 1) was constructed based on the study objectives and relevant help-seeking behavior literature. The initial interview guide was revised after pilot testing with patients with bladder cancer, and these pilot interviews were not included in the final analysis (*n* = 19). Key modifications included separating the original combined question into two independent items addressing symptom discovery and symptom interpretation, and including probes about family awareness of hematuria and what finally prompted participants to seek help. The revised full guide is provided in Supplementary File 1.

**Table 1:** Interview guide.

Interview questions
1. How did you first notice the symptoms that later led to your bladder cancer diagnosis?
2. How did you interpret these symptoms? What did you believe might have caused them?
3. What actions did you take in response to these symptoms before seeking formal medical help?
4. What factors influenced your help-seeking decisions regarding these symptoms?
5. What prompted you to seek medical help?
6. How do you think healthcare professionals should help people experiencing similar symptoms recognize symptoms earlier and seek help promptly?

The face-to-face interviews were conducted by the first author. As a qualified urology nurse, she holds a master's degree, has received specialized training in qualitative methods, and possesses over 10 years of clinical experience in urology. Prior to the interview, the interviewer established rapport with the participants by introducing herself, explaining the research purpose and content, confirming the interview time, and outlining the procedure. Written informed consent was obtained before each interview. Interviews were conducted under two arrangements depending on the interviewer's prior relationship to the participant. When the interviewer was not the participant's attending nurse, interviews were scheduled during breaks in clinical procedures in a private consultation room. When the interviewer was the participant's attending nurse, interviews were conducted only after the nurse-patient relationship had formally ended, typically on the day of hospital discharge, in a neutral private room outside the clinical ward. All interviews were designed to ensure comfort, confidentiality, and voluntary participation. Following the interview guide, the researcher used probing techniques to encourage detailed accounts of patients' symptom experiences, coping behaviors, and help-seeking needs. Non-verbal cues such as gestures, facial expressions, and tone of voice were recorded in field notes along with verbal responses. Interviews lasted 21–43 min (mean 30 min) and were audio-recorded. The interviewer transcribed the audio recordings verbatim and compiled the field notes within 24 hours after each session. No repeat interviews were conducted. The sample size was determined by data saturation, defined as the point during data collection and analysis at which no new data were found that revealed a new category.^23^ In this study, data saturation was confirmed after the initial 17 interviews, two additional interviews were conducted; no new codes relevant to the study aim were identified, and the coding framework remained stable.

### Data analysis

**Fig. 1:**
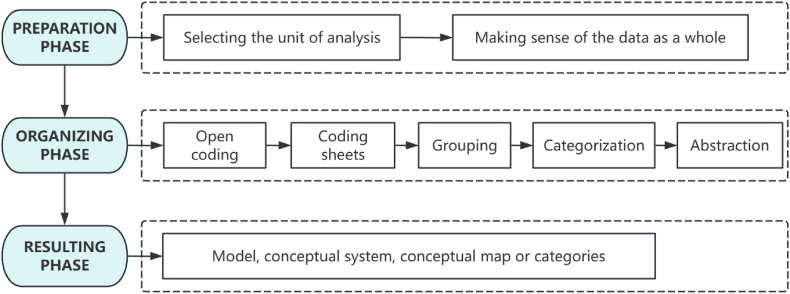
Preparation, organizing and resulting phases in the content analysis process.

Guided by the content analysis approach,^24^ two researchers (the first and second authors) analyzed the data using NVivo 11 software to investigate help-seeking behaviors related to cancer symptoms in patients with bladder cancer. The specific analytical procedure is shown in Fig. 1.

For the preparation phase, the unit of analysis was determined to be meaningful semantic segments from the interview transcripts about help-seeking behavior, and both researchers repeatedly and independently read all transcripts to immerse themselves in the data and gain a holistic understanding of patients' experiences. During the organizing phase, the researchers independently performed line-by-line coding on the initial transcripts to generate initial codes, then met to compare, discuss, and reconcile these codes into a preliminary coding sheet. Through grouping codes with similar meanings and abstracting them into labeled categories, the analysis progressed to examine relationships and hierarchies among categories. Finally, in the resulting phase, key themes encapsulating the core experiences of help-seeking among patients with bladder cancer were formulated through this iterative process. To ensure analytical trustworthiness, the researchers maintained independent work with regular discussions, and any discrepancies were resolved through consensus discussions within the broader research team until final thematic agreement was achieved.

### Assuring rigor

This study employed the criteria of credibility, transferability, dependability, and confirmability to ensure methodological rigor.^25^ The interviewer also participated in some interviewees’ clinical nursing work, establishing trust and rapport with them. However, we also recognized that this dual role could introduce power imbalance and social desirability bias. To mitigate these risks, we implemented the following strategies: interviews were conducted only after the nurse-patient relationship had formally ended, and were typically scheduled on the day of hospital discharge; at the beginning of each interview, the interviewer explicitly stated her role as a researcher, not a nurse, and reassured participants that answers would not affect their care; interviews were held in a neutral private room outside the clinical ward; confidentiality was emphasized repeatedly. Furthermore, the researcher consistently maintained an objective stance throughout the processes of data collection, analysis, and interpretation to enhance credibility. The transferability of the findings was supported by the comprehensive reporting of the study background, participant sampling strategy, inclusion and exclusion criteria, and research methods. To ensure dependability, we retained the following materials as an audit trail: audio recordings of all 19 interviews; anonymized verbatim transcripts; field notes recorded after each interview, documenting the setting, participant affect, and non-verbal cues; coding sheets; and records of team discussions. For example, initially one coder placed “symptoms come and go” under the code “symptom perception.” During team discussion, it was noted that patients mistakenly believed they had recovered after hematuria disappeared, a phenomenon strongly associated with delayed help-seeking and specific to bladder cancer. The team therefore agreed to elevate it to an independent subtheme termed the “illusion of self-healing.” Confirmability was ensured through member checking, in which three participants verified their own statements and confirmed their alignment with the research findings.

## Results

### Demographic characteristics of participants

A total of 19 eligible patients with bladder cancer were enrolled in this study, including 13 males and 6 females. The participants' ages ranged from 34 to 87 years, with a mean of 65.74 years. Regarding educational background, 31.58% of patients had attained a primary school education. Additionally, 89.47% of participants were married, 57.89% resided in rural areas, and 31.58% were employed. A total of 42.11% sought medical help after experiencing symptoms for more than 3 months. The details of patients’ demographic characteristics are presented in Table 2.

**Table 2:** Participant characteristics (*N* = 19).

Demographic characteristics		*n*	%
Sex	Male	13	68.42
	Female	6	31.58
Age, years	< 60	4	21.05
	60–69	7	36.84
	70–79	5	26.32
	≥ 80	3	15.79
Educational level	Primary school	6	31.58
	Junior high school	8	42.11
	Senior high school	2	10.53
	Bachelor	3	15.79
Marital status	Married	17	89.47
	Divorced	1	5.26
	Widowed	1	5.26
Residence	Rural	11	57.89
	Urban	8	42.11
Employment status	Employed	6	31.58
	Unemployed	4	21.05
	Retired	9	47.37
Time of seeking medical help after symptoms	< 1 month	9	47.37
	1–3 months	2	10.53
	> 3 months	8	42.11

### Thematic analysis

**Fig. 2:**
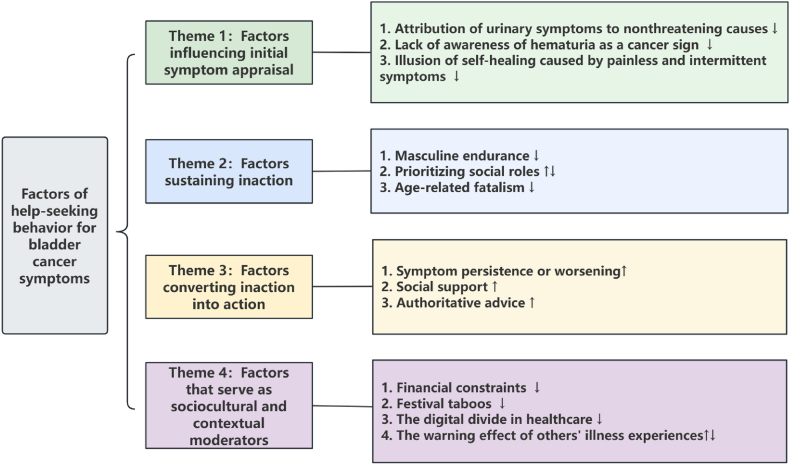
Qualitative thematic summary of factors associated with help-seeking for bladder cancer symptoms. Not a causal model. ↓ indicates delayed help-seeking; ↑ indicates prompted help-seeking; ↑↓ indicates the factor may either prompt or delay help-seeking.

The participant counts reported below are descriptive and non-mutually exclusive, as individual participants could contribute to more than one category or theme. The themes identified through the analysis are shown in Fig. 2. This figure is intended as a thematic summary of associated factors, not as a grounded theory process model or a tested causal pathway. Four major themes were identified: (1) Factors influencing initial symptom appraisal, (2) Factors sustaining inaction, (3) Factors converting inaction into action, (4) Factors that serve as sociocultural and contextual moderators.

#### Theme 1. Factors influencing initial symptom appraisal

This theme describes how participants perceived their symptoms after experiencing symptoms such as hematuria. They generally lacked awareness of the association between hematuria and cancer, often attributing the symptoms to non-threatening causes such as tiredness or aging. Moreover, the painless and intermittent nature of the symptoms created the illusion that they had recovered. These perceptions together led to delays in help-seeking.

##### Attribution of urinary symptoms to non-threatening causes

The primary initial symptoms experienced by patients included hematuria, frequent urination, urgency, and dysuria. Some participants (*n* = 5) attributed these symptoms to benign causes such as diet, fatigue, inflammation, or kidney stones. Counts are non-mutually exclusive because participants could contribute to multiple categories.

*“I thought it was due to eating too many cherry tomatoes—it went away in just one day. After drinking plenty of water, the hematuria was gone the next day.” (P16, male, 76 years old)*

*“I had a fever right before this. After it subsided, I barely rested and went straight back to work, teaching over twelve hours a day, nonstop. So I figured it was probably because I was just exhausted.” (P12, female, 34 years old)*

*“Probably it was inflammation in the urethra.” (P15, male, 67 years old)*

Some patients (*n* = 6) managed their symptoms with medication. Two of these patients delayed seeking help until they developed lower back pain.

*“I just bought some medicine at the pharmacy, the kind that clears heat.” (P8, female, 52 years old)*

*“Later, I felt lower back pain and went to see a doctor, and that was when the tumor was discovered.” (P3, male, 61 years old)*

##### Lack of awareness of hematuria as a cancer sign

None of the participants realized that urinary symptoms such as hematuria, frequency, and urgency might be associated with bladder cancer, nor were they aware that these were typical symptoms of bladder cancer.

*“I had a history of pyelonephritis, so I thought perhaps the bleeding was coming from the ureter; I didn’t think it could be the bladder.” (P18, female, 83 years old)*

Some patients (*n* = 4) recognized that the presence of hematuria was an abnormal bodily signal, but they did not think it was serious enough to be a warning sign of cancer.

*“Last year, while traveling in Yunnan, I was on the flight back when I passed dark red, murky urine [described as resembling stirred mung bean soup]. I immediately thought something was wrong, but did not think it was a tumor.” (P5, male, 69 years old)*

##### Illusion of self-healing caused by painless and intermittent symptoms

Patients developed an illusion of self-healing because the symptoms were both painless and intermittent. The absence of pain removed the urgency to seek help, and the spontaneous disappearance of symptoms after a short time led most patients (*n* = 10) to regard the problem as self-limiting. This pattern repeatedly reinforced the false belief that they had recovered, causing them to underestimate the potential severity.

*“It’s probably just old age; after one episode of bleeding, the next time I peed, it’d be a bit lighter. This whole thing usually lasted two or three days. Then about a month later, the blood showed up again. It wasn’t an everyday thing, just came and went. So I joked with my wife: ‘Feels like I've turned into a woman, getting my monthly visit too!’ [laughed].” (P10, male, 87 years old)*

Additionally, the symptoms typically occurred without pain; some patients (*n* = 4) held the belief that “no pain means no big deal”. This mindset was especially common among older patients, who tended to ignore physical abnormalities that did not interfere with their daily lives.

*“I didn’t feel any pain or discomfort—I could eat, walk fast, and even play mahjong. So I thought it probably wasn’t a big deal.” (P6, female, 74 years old)*

#### Theme 2. Factors sustaining inaction

This theme describes why patients remained inactive after symptom recognition. Masculine endurance and age-related fatalism reinforced delay. However, prioritizing social roles had a dual effect: it usually delayed help-seeking, but for primary caregivers of sick family members, it prompted immediate consultation.

##### Masculine endurance

In this study, masculine endurance was manifested as a psychological and behavioral tendency deeply rooted in gender roles; some male patients (*n* = 7) chose to endure or manage their symptoms on their own first, rather than communicating with their families. Due to the concealability of urinary symptoms, family members were unlikely to detect them without the patient's active disclosure.

*“I didn’t tell my family at first. I have this mindset that I should handle things on my own if possible, so as not to bother others.” (P16, male, 76 years old)*

*“I didn’t tell my son. If I had, he would have made me go to see the doctor earlier.” (P11, male, 53 years old)*

Masculine endurance also reflects men's consideration of the whole family. Concerned that informing their families might cause unnecessary anxiety or distress, and given their children's busy work schedules, they tended to handle their symptoms alone.

*“I kept it from them mainly because I didn’t want them to worry about me.” (P17, male, 80 years old)*

*“We usually handle things on our own. Our kids are quite busy with their work; I don’t want to trouble them.” (P10, male, 87 years old)*

##### Prioritizing social roles

Prioritizing social roles in our study means how patients’ health decisions were guided by their perceived duties to work and family. This factor could either delay or facilitate help-seeking behavior. For some working participants (*n* = 3), work was their primary concern, which prevented them from seeking help immediately.

*“My husband told me to go to the ER, but I said, ‘It's nothing serious.’ I didn’t pay much attention and thought I needed to get this work done first. I figured I'd just go see a doctor over the weekend.” (P14, female, 38 years old)*

*“This time last year I was out of the country for business, so I just put off getting checked out, and that ended up delaying everything for another six months.” (P11, male, 53 years old)*

For the other two male participants, their role as primary family caregivers prompted timely help-seeking. Both had ill wives and stated that they could not afford to fall ill themselves, as their wives depended on them for care. Consequently, they sought help promptly when symptoms arose.

*“My wife has a blood disease; she’s been on steroids for years, which led to osteoporosis. She can hardly manage anything, so I take care of all the housework. I can’t afford to get sick.” (P2, male, 77 years old)*

##### Age-related fatalism

Age-related fatalism refers to the tendency among older patients to attribute illness to the natural process of aging, often accompanied by a sense of resignation or acceptance. In our study, some patients (*n* = 3) did not perceive intermittent, non-bothersome symptoms as a disease threat. Even when a tumor was eventually identified, they accepted it as a natural part of aging.

*“I'm already in my 80s, aren't I? It doesn't matter anymore.” (P18, female, 83 years old)*

*“I’m old, just leave it to fate.” (P10, male, 87 years old)*

Among the 3 patients, an older female patient attributed her diminished motivation to seek help to a sense of cognitive decline, which she perceived as an inevitable part of growing old, reflecting a fatalistic acceptance of aging.

*“I didn’t bother with it. I thought, I’m already this old—when you get old, you just get muddled.” (P6, female, 74 years old)*

#### Theme 3. Factors converting inaction into action

This theme describes factors that eventually prompted patients to seek medical help after a period of inaction. Symptom persistence or worsening, social support from family members, and authoritative advice all played roles in converting inaction into action.

##### Symptom persistence or worsening

Symptom persistence or worsening disrupts patients’ prior tolerance or trivialization and prompts them to seek help. In our study, for some patients (*n* = 3), the progression from intermittent spotting to several consecutive days of hematuria, and from blood at the start of urination to total bloody urine, finally prompted them to take the symptom seriously.

*“At first, there was just a little blood on the toilet paper. Then it increased, and eventually, the entire stream of urine was red. That’s when I knew something was wrong.” (P6, female, 74 years old)*

For others (*n* = 3), the immediate trigger for help-seeking was the development of clot retention, which caused urinary obstruction and urgency.

*“When I peed, clots would come out. Once they passed, I felt fine, no more bloating or pain. But when nothing came out, the pain and pressure were awful [expletive omitted]. So I had to sit on the toilet like a woman to pee. Couldn’t even sleep through the night. This is too much; I can’t bear it anymore.” (P4, male, 73 years old)*

*“I couldn’t pee at all, and it totally freaked me out. So I went to see a doctor right away.” (P10, male, 87 years old)*

##### Social support

Social support from immediate family members or friends often served as a critical catalyst that prompted patients to move from delay to action. For three patients, it was their spouse or adult children who first noticed the symptoms and promptly arranged for them to seek medical help.

*“My wife noticed traces of blood in the toilet and asked me about it. I told her I’d had blood in my urine for a few days. She immediately called our daughter, and they took me to see a doctor.” (P17, male, 80 years old)*

Support from friends was identified as an effective catalyst for medical help-seeking. Two female patients received decisive support after confiding their symptoms to friends; one received direct accompaniment to the hospital, while the other was met with daily reminders and strong encouragement.

*“My friend found out and then urged me to get tested, at noon, in the evening, and at night, until I went to the clinic. That’s why I was able to see the doctor so quickly.” (P12, female, 34 years old)*

##### Authoritative advice

Professional guidance from healthcare providers also served as a triggering factor. For some patients (*n* = 3), the long-standing neglect or misattribution of hematuria was only truly disrupted after receiving a warning from an authoritative source. Authoritative figures could be trusted medical professionals.

*“There was an old doctor back in my hometown, a really, really good one. He was always so busy. But he told me to come to a big hospital for a check-up.” (P13, male, 62 years old)*

Authoritative advice also came from family members with a medical background, as their professional knowledge made their advice particularly influential.

*“Since my nephew is a physician, he told me to go to the hospital for a check-up.” (P7, male, 62 years old)*

#### Theme 4. Factors that serve as sociocultural and contextual moderators

This theme captures contextual elements that shaped the strength or direction of factors influencing help-seeking. Festival taboos, financial constraints, and the digital divide in healthcare amplified delays. The warning effect of others’ illness experiences had a dual influence, either prompting action or reinforcing delay.

##### Financial constraints

Eight participants considered financial factors when seeking medical help. Cost presented a significant barrier, particularly among low-income individuals and those dependent on family financial support.

*“The cost is just too much. My son has two kids, and they’re both still in school. And I'm not earning any money now.” (P4, male, 73 years old)*

*“Our pension is only a few hundred yuan, so we rely entirely on our son.” (P19, female, 72 years old)*

Limited financial resources had to be prioritized for basic living expenses rather than healthcare.

*"Our house was sinking and practically crumbling down. We had to tear it down and rebuild, which took over two years and left us with significant debt. My pension is only 2000 CNY a month, so I kept putting off seeing a doctor." (P13, male, 62 years old)*

##### Festival taboos

In traditional Chinese culture, specific festivals are considered inauspicious for seeking medical treatment, notably the Spring Festival (Chinese New Year). Consequently, individuals often avoid going to the hospital during this period. In our study, two patients delayed seeking medical help during the Spring Festival.

*“It's already the end of the year. Later on, I'd just put it off till after Spring Festival.” (P1, male, 61 years old)*

One of these patients, despite having been hospitalized for evaluation, requested discharge as the Spring Festival approached. She explained:

*“I was hospitalized for tests, but before I could even have the cystoscopy, it was almost the New Year [Spring Festival]. I said I wanted to go home; I refused to stay in the hospital.” (P6, female, 74 years old)*

##### The digital divide in healthcare

In this study, several older participants reported varying degrees of difficulty using smart devices, particularly those living in rural areas. Many either did not own such devices or, even if they did, could not use them effectively.

*“I just can’t figure out these smartphones. Ours are basic phones for seniors; you can't make appointments on them.” (P10, male, 87 years old)*

Due to the digital divide, they relied on their children for assistance with medical visits, which to some extent delayed their help-seeking behavior.

*“Last time my son didn’t come, I had no idea where to go. Everything was operated through the phone and the machine. We didn't get much schooling.” (P13, male, 62 years old)*

*“My daughter-in-law found out online which senior specialist was good and booked me an expert appointment. I couldn't have done it myself.” (P6, female, 74 years old)*

##### The warning effect of others’ illness experiences

The warning effect refers to how witnessing or learning about the illness experiences of family members or friends can shape one's own health perceptions and help-seeking behavior. In this study, some participants reported that seeing close relatives or acquaintances develop serious diseases, particularly cancer, made them more aware of their own health risks and prompted them to take action.

*“My wife has breast cancer, and my son’s father-in-law had back surgery. Seeing so many people around me fall ill, I feel I should be more concerned about myself.” (P9, male, 68 years old)*

*“We’re quite scared in this regard, because my mother-in-law passed away from a tumor two years ago.” (P12, female, 34 years old)*

However, for some participants, others’ illness experiences did not prompt action. Instead of becoming more vigilant, they engaged in wishful thinking.

*“A close former colleague who lived upstairs from me was hospitalized for cancer and died within 17 days, but I didn't take it too seriously. I kept thinking that something like that wouldn't happen to me – just wishful thinking.” (P16, male, 76 years old)*

## Discussion

### Main findings

The four themes describe the factors shaping the experiences of Chinese patients with bladder cancer from symptom onset to medical help-seeking. In factors influencing initial symptom appraisal, patients generally lacked awareness that hematuria could be a sign of cancer, often attributing symptoms to tiredness, aging, and other benign causes. The painless and intermittent nature of symptoms created an illusion of self-healing. Factors sustaining inaction, including masculine endurance, prioritizing social roles, and age-related fatalism, kept patients from acting. The emergence of factors converting inaction into action prompted patients to finally seek medical help. Contextual moderators influenced the strength of the above associations. These findings indicate that help-seeking behavior among Chinese patients with bladder cancer is shaped by cognitive, cultural, social, and structural factors.

This study found that most Chinese participants with bladder cancer, including highly educated individuals such as a university teacher and a bank employee, did not associate hematuria with bladder tumors, but rather attributed it to food, fatigue, or inflammation. This may partly reflect the broader Chinese cultural context where cancer is often met with fear and stigma.^26^ Only a few patients instinctively recognized that hematuria indicated a serious problem. This suggests that public awareness of hematuria as an early warning sign of bladder cancer is low. These findings are similar to those of studies among patients with cervical cancer^27^ and colorectal cancer,^28^ which suggest that cancer knowledge is a cornerstone of help-seeking behavior. Notably, unlike the study by Fennell et al.,^29^ we found that the relationship between education level and symptom perception may not be straightforward, knowledge gaps were observed across educational backgrounds in this sample, suggesting that the issue was not confined to participants with lower educational attainment. This may reflect that the issue is not merely a lack of personal knowledge, but also involves sociocultural norms that normalize or obscure the seriousness of early symptoms.

In addition, the intermittent and painless nature of hematuria appeared to reduce perceived urgency in this study. Most patients misinterpreted the symptom as a problem that could resolve itself. According to the “timeline” dimension of the Common-Sense Model of Self-Regulation,^30^ individuals’ perception of a symptom includes subjective judgments and objective measurements of its onset speed, duration, and rate of decline. This determines whether they view the problem as short-term or long-term, thereby influencing their help-seeking behavior. Therefore, promoting early help-seeking for bladder cancer symptoms may not be effective if campaigns rely solely on general public messages such as “be alert to hematuria.” Health education campaigns could explicitly emphasize that painless hematuria is the most common first symptom of bladder cancer, and that even if it occurs only once or disappears quickly, medical evaluation is still necessary.

Unlike symptom perception in the initiation phase, masculine endurance creates a tacit tolerance of symptoms. The incidence of bladder cancer in men is approximately three to four times higher than in women,^31^ and the disease predominantly affects the elderly population. Elderly men, as the group most commonly affected, tend to exhibit masculine endurance under the influence of traditional gender norms. This theme has also been observed in studies of prostate cancer patients.^32^ However, this study further reveals that within the Chinese cultural context, masculine endurance is not merely a manifestation of individual personality traits but is reinforced by intergenerational altruism: patients actively choose to conceal their symptoms to protect their adult children from anxiety.

Prioritizing social roles is an important factor influencing help-seeking behavior. Employed patients often delay seeking medical care due to work commitments. In this study, participants’ accounts suggested that work-related delays were intertwined with a sense of personal obligation and social expectations, which may resonate with the theme of competing social responsibilities identified in systematic reviews of cancer help-seeking behavior.^33^ By contrast, family responsibility accelerated help-seeking in this study. Unlike patients with breast cancer in Oman who delayed seeking care because of prioritizing child care,^34^ or those in India who delayed due to family obligations,^35^ patients in this study believed that only by ensuring their own health first could they better fulfill their family responsibilities, thereby prompting timely help-seeking.

The perception of age-related fatalism may contribute to viewing bladder cancer as a natural part of aging rather than a medical event, which could reduce the perceived obligation to seek care. Such fatalism is consistent with previous reports on cancer in older adults,^36^ but our study reveals another specific dimension: for some older Chinese patients, fatalism intertwines with a sense of cognitive decline. The combination of physical symptoms and perceived mental dullness further weakens their agency to seek medical help.

This study found that symptom persistence or worsening, social support, and authoritative were described by participants as triggers for help-seeking in patients with bladder cancer. Our findings suggest that social support may be a key factor shaping patients' help-seeking behavior. Evidence indicates that even initially avoidant patients may show an increased likelihood of moving toward help-seeking when supported by family members or friends.^37^ Bladder cancer mainly affects older adults, with a median age at diagnosis of approximately 73 years in US registry data.^38^ Therefore, within the Chinese cultural context, where older adults predominantly rely on their adult children for help-seeking, the involvement of family becomes a critical accelerator for timely help-seeking. Authoritative warnings redefine symptoms by providing a credible, external framework, thereby breaking patients’ prior misattribution. When a credible source labels the symptom and recommends action, patients are more likely to seek help promptly.

This study also identified a range of situational constraints and resource availability factors that permeate the entire help-seeking process. These factors were not described by participants as direct triggers or barriers to help-seeking behavior, but rather emerged as contextual conditions that shape the direction or strength of participants' decisions, thereby influencing patients’ help-seeking behavior.

Financial constraints are a significant concern for many individuals with bladder cancer, which represents the most expensive malignancy to treat on a per-patient basis among the elderly.^39^ This is consistent with previous research indicating that the financial burden is a barrier to help-seeking for cancer symptoms.^40^^,^^41^ For Chinese patients with bladder cancer, the decision to seek care was not merely a medical one but a stark financial calculation, forcing a trade-off between personal health and family economic stability. Festival-related beliefs emerged in this sample as a culturally shaped contextual factor. The Spring Festival is endowed with meanings such as family reunion and good fortune, yet it has also fostered a social convention of avoiding medical visits during this period. According to the Theory of Social Norms,^42^ descriptive norms are formed by observing others' behaviors and provide individuals with behavioral references. When the practice of “not seeking medical care during the Spring Festival” is repeatedly imitated and perpetuated over time, it gradually solidifies into a social habit, thereby influencing patients’ timing of seeking medical help. The digital divide constitutes a structural barrier for elderly patients, attributable to the complexity of operating digital devices and a slower acceptance of new technologies.^43^ Research indicates that individuals aged 65 and above are particularly susceptible to digital exclusion.^44^ To address this challenge, digital solutions should include the development of senior-friendly mobile interfaces and dedicated application features.^45^

### Implications for nursing practice and research

#### Enhancing symptom recognition and risk awareness

Common intervention forms for improving patients' tumor-related knowledge and awareness include educational sessions, films and booklets, and web-based decision aids.^46^ Public education should explicitly emphasize that painless, intermittent hematuria may be a sign of bladder cancer. Health campaigns should counter common misattributions and the illusion of self-healing caused by symptom intermittency. In China, the family doctor contracting service is provided by family doctors and nurses to ensure continuity, safety, and effective basic medical service, enabling them to deliver more comprehensive health education to patients.^47^

Given the reported effectiveness of nurse-led healthcare services at the primary-secondary care interface,^48^ such approaches may be useful for enhancing symptom awareness in this population.

#### Overcoming cultural-psychological barriers

To address masculine endurance enabled by symptom concealability, interventions should encourage symptom disclosure and family involvement. Family members, especially adult children, can be educated to actively inquire about urinary symptoms in older relatives and to provide timely encouragement for medical consultation. For elderly patients living apart from their children, a “family–community–hospital” linkage mechanism might help ensure continuity of care. Nurse-led educational activities using visual comparison charts and repeated simple messages could help older patients distinguish between normal aging and disease.

#### Enhancing the accessibility of catalysts for help-seeking

Healthcare systems can facilitate symptom monitoring by providing patients with simple symptom diaries or verbal guidance on recognizing when intermittent hematuria becomes persistent or worsens. Strengthening social support may involve educating family members, especially adult children, to actively inquire about urinary symptoms in older relatives and to encourage timely help-seeking. Brief reminders from a family doctor during routine visits or a concerned call from a medically trained relative may prompt patients to seek help. In addition, peer support groups organized online or offline, where patients and family members share their experiences and coping strategies, may reinforce the decision to consult a healthcare provider.

#### Addressing situational constraints and resource limitations

To reduce the suppressive effect of festival taboos, such as avoiding care during the Spring Festival, pre-holiday health campaigns could normalize timely medical consultation. For patients with financial concerns, healthcare providers should proactively inform them about relevant policies like medical insurance and charitable assistance. To address the digital divide, we recommend implementing simple offline appointment services and volunteer-assisted digital training, along with improving the digital health competencies of healthcare professionals.^49^

### Limitations

This study is subject to three main limitations. First, although in-depth interviews were conducted with 19 patients possessing diverse disease experiences and demographic characteristics, yielding relatively rich qualitative data and initially reaching data saturation, the sample size may still be insufficient. Second, because this was a single-center qualitative study, transferability to other settings may be limited. Third, this study relied on retrospective self-reports, which are subject to hindsight bias. Patients may reinterpret their initial symptom reactions after a cancer diagnosis. Although we used open-ended and non-leading questions to minimize this bias, some degree of recall distortion remains possible. Future prospective studies could recruit patients presenting with hematuria or other high-risk symptoms to better capture real-time help-seeking processes, and multi-center collaborations with stratified sampling based on geographic location, hospital type, and cultural background would further enhance the information power of the results.

## Conclusions

The study describes the factors influencing help-seeking behavior for cancer symptoms among Chinese patients with bladder cancer. Patients did not recognize hematuria as a possible cancer sign. They attributed urinary symptoms to benign causes like tiredness or aging, and the painless, intermittent nature of the symptoms created a false sense of self-healing. These perceptions collectively delayed help-seeking. Among the identified factors, masculine endurance, age-related fatalism, and festival-related beliefs emerged as salient, culturally shaped influences in this sample. Therefore, stratified, family-involved, digitally assisted, and culturally sensitive interventions could be considered. Through multi-level collaborative efforts, public risk awareness of hematuria may be enhanced, help-seeking for symptoms may be facilitated, and earlier presentation to care may be supported.

## CRediT authorship contribution statement

**Bin Wang:** Writing-original draft, Validation, Software, Methodology, Investigation, Formal analysis, Data curation, Conceptualization. **Jun Zou:** Writing-review & editing, Software, Methodology, Formal analysis, Data curation, Conceptualization. **Lingling Gao:** Writing-review & editing, Formal analysis, Conceptualization. **Fang Lou**: Writing-review & editing, Methodology, Formal analysis. **Ziyi Qi:** Methodology, Writing-review & editing, Formal analysis. **Yun Dai:** Writing-review & editing, Supervision, Resources, Project administration, Funding acquisition, Methodology, Conceptualization. All authors have read and approved the final manuscript.

## Ethics statement

The study received institutional ethics approval from The First Affiliated Hospital of Zhejiang University School of Medicine (Approval No. 2024-989) and was conducted in accordance with the 1964 Helsinki Declaration and its later amendments or comparable ethical standards. All participants provided written informed consent.

## Data availability statement

The datasets generated and analyzed are not publicly available due to their sensitive nature; disclosure could compromise participant privacy and breach the confidentiality assurances given in the informed consent process.

## Declaration of generative AI and AI-assisted technologies in the writing process

During the preparation of this work, the authors used Grammarly in order to improve language editing and proofreading. After using this tool, the authors reviewed and edited the content as needed. The authors take full responsibility for the content of the publication.

## Funding

This work was supported by the Medical and Health Research Project of Zhejiang Province (Grant No. 2025KY826). The funder had no role in the study design; data collection, analysis, or interpretation; preparation of the manuscript; or the decision to submit the article for publication.

## Declaration of competing interest

The authors declare no conflicts of interest.
