# Supplementary material for: Factors associated with help-seeking for urinary symptoms among Chinese patients with bladder cancer: A qualitative study
Source: Asia Pac J Oncol Nurs. 2026 Jul 1;13:101001. doi: 10.1016/j.apjon.2026.101001 (PMC13382044; doi:10.1016/j.apjon.2026.101001)
Supplement: Multimedia component 1 [file mmc1.doc]

**Supplementary File 1**

The initial guide was developed based on literature review and clinical experience, consisting of five questions:

1. How did you discover the bladder tumor, what symptoms appeared, and how did you perceive these symptoms?
2. What difficulties or distress did these bladder tumor‑related symptoms cause you?
3. How did you manage these bladder tumor‑related symptoms?
4. What factors influenced your management of these symptoms?
5. What needs do you have regarding symptom management of bladder tumor, and what support can healthcare professionals provide?

**Problems identified during pilot testing** **(with two patients)**

| Problem | Evidence from pilot interviews |
| --- | --- |
| 1. **Question 1**   **combined three distinct topics** (discovery, symptoms, perception) | Participants often answered only the first part (“I saw blood in urine”) and omitted attribution/perception unless repeatedly prompted. The compound question was cognitively overloaded. |
| **B. Question 2 (“difficulties/distress”) diverted focus away from help‑seeking** | Participants responded with descriptions of physical pain or emotional worry, but did not naturally discuss **delay or help‑seeking decisions**. The concept of “associated factors of help‑seeking behavior” was not captured. |
| **C. Question 3 (“how did you manage symptoms”) was vague** | Some participants answered “I went to the doctor” (already seeking help), while others answered “I did nothing” or “I rested more.” The term (management) was interpreted inconsistently, and the question did not probe for **actions before formal medical help**. |
| **D. No explicit question about triggers for help‑seeking** | The guide asked about “influencing factors” (Q4) but not directly “What finally prompted you to seek medical help?” Triggers such as symptom aggravation or family urging were only mentioned if participants spontaneously offered them. |
| **E. No exploration of family awareness or symptom concealability** | One pilot participant spontaneously said: “My wife didn’t know about the blood – only I could see it. When she found out, she pushed me to go.” This revealed an important factor (symptom privacy, family knowledge) not covered in any original question. |

**Specific revisions made after pilot testing**

| Problem | Revision | How it appears in the final guide |
| --- | --- | --- |
| **A (compound Q1)** | Split into **two separate questions**: one for symptom discovery, one for interpretation. | Final Q1: “How did you first notice the symptoms that later led to your bladder cancer diagnosis?” Final Q2: “How did you interpret these symptoms? What did you believe might have caused them?” |
| **B (Q2 off‑target)** | **Deleted** Q2 entirely (“difficulties/distress”) because it did not address help-seeking. | Not present in final guide. |
| **C (vague “management” Q3)** | **Replaced** Q3 with a more specific question about **actions before formal medical help**, with added timeline prompts. | Final Q3: “What actions did you take in response to these symptoms before seeking formal medical help?” |
| **D (no explicit trigger question)** | **Added a new question** directly asking what finally prompted help-seeking. | Final Q5: “What prompted you to seek medical help?” |
| **E (no family awareness probe)** | Added a **probe** about family members’ awareness of hematuria, following Q4. | After final Q4, if not spontaneously mentioned: “Did anyone in your family know about the blood in your urine at the time? If not, why did you choose not to tell them? If yes, how did they react, and did that affect your decision to see a doctor?” |
| **Also: Q4 wording refined** | Original Q5 referred to “management of symptoms”; revised to explicitly reference **help-seeking decisions**. | Final Q4: “What factors influenced your help-seeking decisions regarding these symptoms?” |
| **Q5 retained but reworded** | Original Q5 asked about “symptom management needs”; reworded to focus on **early recognition and timely help-seeking** as per study aim. | Final Q6: “How do you think healthcare professionals should help people experiencing similar symptoms recognize symptoms earlier and seek help promptly?” |
